# Supplementary material for: Structural mechanism of GTPase-powered ribosome-tRNA movement
Source: Nat Commun. 2021 Oct 11;12:5933. doi: 10.1038/s41467-021-26133-x (PMC8505512; doi:10.1038/s41467-021-26133-x)
Supplement: Supplementary file 3 — Description of Additional Supplementary Files [file 41467_2021_26133_MOESM3_ESM.pdf]

Description of Additional Supplementary Files

File Name: Supplementary Movie 1
Description: **GTPpase-powered tRNA translocation step visualized by cryo-EM.**
The movie illustrates how GTP hydrolysis by the translational GTPase EF-G results in large-scale molecular movement driving forward tRNA movement on the ribosome. The animation is based on the experimental structures, except the pre-translocation state with EF-G-GTP, which was modeled based on the H1–EF-G–GDP–Pi structure.
