# Supplementary material for: Structural mechanism of GTPase-powered ribosome-tRNA movement
Source: Nat Commun. 2021 Oct 11;12:5933. doi: 10.1038/s41467-021-26133-x (PMC8505512; doi:10.1038/s41467-021-26133-x)
Supplement: Supplementary file 5 — Reporting Summary [file 41467_2021_26133_MOESM5_ESM.pdf]

nature research

Corresponding author(s): Niels Fischer
Marina V. Rodnina

Last updated by author(s): Aug 27, 2021

Reporting Summary

Nature Research wishes to improve the reproducibility of the work that we publish. This form provides structure for consistency and transparency in reporting. For further information on Nature Research policies, see our Editorial Policies and the Editorial Policy Checklist.

Statistics

For all statistical analyses, confirm that the following items are present in the figure legend, table legend, main text, or Methods section.

n/a	Confirmed	
☐	☒	The exact sample size (*n*) for each experimental group/condition, given as a discrete number and unit of measurement
☐	☒	A statement on whether measurements were taken from distinct samples or whether the same sample was measured repeatedly
☐	☒	The statistical test(s) used AND whether they are one- or two-sided<br>*Only common tests should be described solely by name; describe more complex techniques in the Methods section.*
☒	☐	A description of all covariates tested
☒	☐	A description of any assumptions or corrections, such as tests of normality and adjustment for multiple comparisons
☐	☒	A full description of the statistical parameters including central tendency (e.g. means) or other basic estimates (e.g. regression coefficient) AND variation (e.g. standard deviation) or associated estimates of uncertainty (e.g. confidence intervals)
☒	☐	For null hypothesis testing, the test statistic (e.g. *F*, *t*, *r*) with confidence intervals, effect sizes, degrees of freedom and *P* value noted<br>*Give P values as exact values whenever suitable.*
☐	☒	For Bayesian analysis, information on the choice of priors and Markov chain Monte Carlo settings
☒	☐	For hierarchical and complex designs, identification of the appropriate level for tests and full reporting of outcomes
☒	☐	Estimates of effect sizes (e.g. Cohen's *d*, Pearson's *r*), indicating how they were calculated

Our web collection on statistics for biologists contains articles on many of the points above.

Software and code

Policy information about availability of computer code

Data collection	Fluorescence data was collected using Pro-Data SX 2.5.1852.0 (Applied Photophysics) for stopped-flow experiments. Liquid scintillation data was collected using QuantaSmart 4.00 (Perkin Elmer). Cryo-EM data collection was performed using software EPU 2.3 (ThermoFisher) and CETCORPLUS 4.6.9 (CEOS). All software is commercially available.
Data analysis	Analytical fit of fluorescence traces and reaction time courses was performed in GraphPad Prism v8. All software is commercially available. All software used for Cryo-EM data analysis has been described in Methods and is publicly available free-of-charge: MCOR2, CTFFIND-4.1, GAUTOMATCH 0.56, RELION 3.1, Coot 0.8.9.2-0.9.3, PHENIX 1.16-3549, iSOLDE 1.1.0, UCSF ChimeraX 1.2, GROMACS 2018.8.

For manuscripts utilizing custom algorithms or software that are central to the research but not yet described in published literature, software must be made available to editors and reviewers. We strongly encourage code deposition in a community repository (e.g. GitHub). See the Nature Research guidelines for submitting code & software for further information.

Data

Policy information about availability of data

All manuscripts must include a data availability statement. This statement should provide the following information, where applicable:

- Accession codes, unique identifiers, or web links for publicly available datasets
- A list of figures that have associated raw data
- A description of any restrictions on data availability

The cryo-EM maps/associated coordinates of atomic models generated in this study have been deposited in the Electron Microscopy Data Bank/Protein Data Bank under the following accession codes: EMD-13458/7PJS (C state); EMD-13459/7PJT (H1); EMD-13460/7PJU (H2); EMD-13461/7PJV (H1–EF-G–GDP–Pi); EMD-13462/7PJW (H2–EF-G–GDP–Pi); EMD-13463/7PJX (H1–EF-G–GDP); EMD-13464/7PJY (CHI1–EF-G–GDP); EMD-13465/7PJZ (CHI2–EF-G–GDP). The cryo-EM micrographs and particle images generated in this study have been deposited in the EMPIAR database with accession code EMPIAR-10792. Raw data of the kinetic

analysis (Fig. 2a and Supplementary Fig. 2a-d) generated in this study are provided in the Source Data file. The atomic coordinates used in this study are available in the Protein Data Bank under accession codes: 3J9Z; 3JCJ; 4AQY; 4V5F; 4V6D; 4V85; 4V8D; 4V8O; 4V90; 4V9H; 4V9K; 4V9O; 4V9P; 4W29; 5AFI; 5LZD; 5OT7; 5UYL; 5UYM; 5YUM; 6O9K; 6WD2; 6WD6; 6WD8; 6YSS. The authors declare that all other data supporting the findings of this study are available within the paper and its supplementary information files.

Field-specific reporting

Please select the one below that is the best fit for your research. If you are not sure, read the appropriate sections before making your selection.

- [x] Life sciences
- [ ] Behavioural & social sciences
- [ ] Ecological, evolutionary & environmental sciences

For a reference copy of the document with all sections, see nature.com/documents/nr-reporting-summary-flat.pdf

Life sciences study design

All studies must disclose on these points even when the disclosure is negative.

Sample size	Sample size was determined using the confidence level criterion. The number of datapoints was chosen to ensure that the precision of the fit is below 1%, which for each technical replicate in the stopped-flow was 2000 for mRNA translocation and 5000 for phosphate release experiments.
Data exclusions	No data were omitted.
Replication	All biochemical experiments have been repeated as independent experiments, as indicated in the figure legends and/or Methods
Randomization	Does not apply to our study, because we do not separate data into treatment groups.
Blinding	Does not apply to our study, because we avoid bias by using all data for analysis

Reporting for specific materials, systems and methods

We require information from authors about some types of materials, experimental systems and methods used in many studies. Here, indicate whether each material, system or method listed is relevant to your study. If you are not sure if a list item applies to your research, read the appropriate section before selecting a response.

Materials & experimental systems

n/a	Involved in the study	
[x]	[ ]	Antibodies
[x]	[ ]	Eukaryotic cell lines
[x]	[ ]	Palaeontology and archaeology
[x]	[ ]	Animals and other organisms
[x]	[ ]	Human research participants
[x]	[ ]	Clinical data
[x]	[ ]	Dual use research of concern

Methods

n/a	Involved in the study	
[x]	[ ]	ChIP-seq
[x]	[ ]	Flow cytometry
[x]	[ ]	MRI-based neuroimaging
